# Characterization and Compatibility Studies of Different Rate Retardant Polymer Loaded Microspheres by Solvent Evaporation Technique: *In Vitro-In Vivo* Study of Vildagliptin as a Model Drug

**DOI:** 10.1155/2015/496807

**Published:** 2015-11-12

**Authors:** Irin Dewan, Swarnali Islam, Md. Sohel Rana

**Affiliations:** ^1^Department of Pharmacy, Jahangirnagar University, Savar 1342, Dhaka, Bangladesh; ^2^Department of Pharmacy, University of Asia Pacific, Dhanmondi, Dhaka 1209, Bangladesh

## Abstract

The present study has been performed to microencapsulate the antidiabetic drug of Vildagliptin to get sustained release of drug. The attempt of this study was to formulate and evaluate the Vildagliptin loaded microspheres by emulsion solvent evaporation technique using different polymers like Eudragit RL100, Eudragit RS100, Ethyl cellulose, and Methocel K100M. *In vitro* dissolution studies were carried out in 0.1 N HCl for 8 hours according to USP paddle method. The maximum and minimum drug release were observed as 92.5% and 68.5% from microspheres, respectively, after 8 hours. Release kinetics were studied in different mathematical release models to find out the linear relationship and release rate of drug. The SEM, DSC, and FTIR studies have been done to confirm good spheres and smooth surface as well as interaction along with drug and polymer. In this experiment, it is difficult to explain the exact mechanism of drug release. But the drug might be released by both diffusion and erosion as the correlation coefficient (*R* ^2^) best fitted with Korsmeyer model and release exponent (*n*) was 0.45–0.89. At last it can be concluded that all *in vitro* and *in vivo* experiments exhibited promising result to treat type II diabetes mellitus with Vildagliptin microspheres.

## 1. Introduction

Vildagliptin is a potent, selective, and orally active dipeptidyl peptidase-4 (DPP-4) inhibitor, which prevents inactivation of incretion hormones by inhibiting DPP-4. It has been shown to be an effective and safe option for better glycemic control in a wide range of T2DM patients and has demonstrated HbA1C lowering potential when given as monotherapy or in combination with other OADs, without weight gain and minimal hypoglycemia [1]. Drugs like sulfonylureas, meglitinides, and insulin are associated with weight gain and hypoglycemia; thiazolidinediones (TZDs) cause weight gain and possibly peripheral edema. Metformin and a-glucosidase inhibitors are associated with gut-related side effects. Additionally, the impact of different drugs, even within a single class, on the risk of long-term vascular complications has recently come under scrutiny [2]. Its biological half-life is 1 to 3 hrs as a consequence; it requires repeated administration to keep plasma concentration. This causes bother to the patient and also leads to fluctuations in plasma drug concentration so that it might reduce the therapeutic effect. But advanced controlled release forms enhance patient compliance by reducing frequency of dosing. Therefore, development of Vildagliptin sustained release dosage forms is desirable to achieve a more effective therapy avoiding the large fluctuations in drug concentration and consequently reduction in adverse effects and to reduce the need of several administrations.

Microspheres constitute an important part of this particulate drug delivery system by virtue of their small size and efficient carrier characteristics. However, the success of this novel drug delivery system is limited due to their short residence time at the site of absorption. It would therefore be advantageous to have means for providing intimate contact of the drug delivery system with absorbing gastric mucosal membranes. Along with a range of methods developed designed for formulation of microspheres, emulsion solvent evaporation technique is one of the typically extensively used ones because of its simplicity of fabrication devoid of compromising the action of drug [3]. This method facilitates altering the liquids to solids, by considering the colloidal and surface properties, as long as there is environmental protection, and controlling the liberate distinctiveness of unlikely coated materials. This has been made by developing the new drug entities, discovering of new polymeric materials that are appropriate for prolonging the drug release and safety and improvement in therapeutic efficacy. In general the size of the microencapsulated products is considered as larger than 1 micrometer and up to 1000 micrometers in diameter [4].

Ethyl cellulose, a nonbiodegradable and biocompatible polymer, one of the extensively studied encapsulating materials for the controlled release of pharmaceuticals, was preferred as the retardant material. Methacrylate copolymers (Eudragits) have recently received increased consideration for modified dosage forms because of their inertness, solubility in relatively nontoxic solvents, and availability of resins with different properties. In the present investigation Eudragit RL is used as a rate retardant polymer. Eudragit RL is a water insoluble polymer which is widely used as a wall material for controlled release microparticles. The permeability of Eudragit RS and Eudragit RL in aqueous media is due to the presence of quaternary ammonium groups in their structure; Eudragit RL has a greater proportion of these groups and as such is more permeable than Eudragit RS [5].

The primary view of the present effort was to prepare and estimate oral controlled release microparticulate drug delivery system of Vildagliptin using different polymers by water-in-oil emulsion solvent diffusion method by means of high entrapment capacity and extended release. Moreover, such small single units enable more reproducible dispersion throughout the gastrointestinal tract leading to reduction of drug release variations and improved bioavailability. Multiple-unit system generally disperses freely in the gastrointestinal fluids, maximizes absorption, minimizes side effects, and reduces inter- and intrapatient variability.

## 2. Materials and Methods

### 2.1. Materials

Materials were Vildagliptin as donation sample from Eskayef Bangladesh Limited, Ethyl cellulose (Colorcon Asia Pvt. Limited, India), Eudragit RS100 (Evonik, Germany), Eudragit RL100 (Evonik, Germany), magnesium stearate (Merck, Germany), Ethanol (Merck, Germany), Dichloromethane (Merck, Germany), light liquid paraffin, Tween 80 (Merck, Germany), cyclohexane (Merck, Germany), sodium hydroxide (Merck, Germany), potassium dihydrogen phosphate (Merck, Germany), *n*-hexane (Merck, Germany), and so forth.

### 2.2. Method

#### 2.2.1. Preparation of Vildagliptin Microspheres by Emulsion Solvent Evaporation Technique

**Figure 1:**
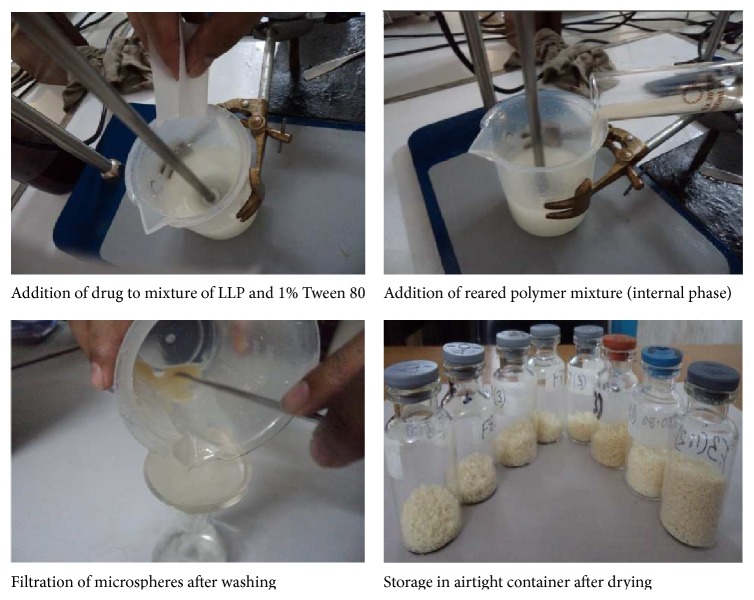
Schematically presenting the steps of microspheres prepared by emulsion solvent evaporation method.

The microspheres were prepared according to Table 1 by solvent evaporation method. The process was initiated from dispersion of Vildagliptin in 70 mL of light liquid paraffin (LLP) using 1% Tween 80. At first, LLP was emulsified in plastic beaker with Tween 80 for few minutes with the help of stirrer at 500 rpm. By this time the polymer solution (internal phase) was prepared by dissolving properly weighed polymer(s) in combination of Ethanol and Dichloromethane at a ratio of 5 : 5 in volumetric flask. Then appropriately weighed drug was added in the internal phase slowly and stirred for 20–30 minutes. After proper mixing prepared polymeric phase was added dropwise to the external phase. Stirring was performed for 2.5 hours. After stirring, the microspheres were decanted and washed by *n*-hexane and allowed to dry in natural air. The microspheres were transferred to glass vials and placed in the desiccators for further experiment as shown in Figure 1.

### 2.3. Preparation of Standard Curve of Pure Vildagliptin

At first 20 mg of Vildagliptin was taken in 100 mL volumetric flask and dissolved in 100 mL 0.1 N HCl and taken into sonicator for 10 minutes. Then 10 mL of the previous solution was taken in another 100 mL volumetric flask and diluted up to 100 mL with 0.1 N HCl and kept as stock solution. Then 1 mL, 2 mL, 3 mL, 4 mL, 5 mL, 6 mL, 7 mL, 8 mL, 9 mL, and 10 mL of stock solution were taken in ten different 10 mL volumetric flasks and diluted all up to 10 mL with 0.1 N HCl. Thus the concentrations of solution ranging from 2 *μ*g/mL to 20 *μ*g/mL were obtained. Finally different concentrations solutions were analyzed by spectrophotometry at 210 nm.

### 2.4. Assay Methods of Prepared Microspheres by Emulsion Solvent Evaporation Technique

Approximately 50 mg of Vildagliptin microspheres was taken in 100 mL volumetric flask and dissolved with minimum quantity of Ethanol. After that 25 mL of 0.1 N HCl was added and sonicated for 30 minutes to make clear solution. Then the solution was finally filtered. Absorbance value was determined using UV spectrophotometer at wavelength of 210 nm. Using the absorbance value, the amount of Vildagliptin entrapped was determined with the help of standard curve. Percent drug loading and drug entrapment efficiency were calculated by using the following equation:

(1) %  drug  loading=Actual  drug  loadingWeighed  quantity  of  microspheres×100,Drug  entrapment  efficiency  %=Actual  drug  loadingTheoretical  drug  loading×100.

### 2.5. *In Vitro* Release Study of Vildagliptin Microspheres Prepared by Emulsion Solvent Evaporation Technique

*In vitro* dissolution study was performed in paddle type dissolution apparatus. At first 900 mL of 0.1 N HCl was used as dissolution media, paddle speed was 100 rpm, and temperature was maintained fixed at 37°C. Approximately 20 mg equivalent amount of Vildagliptin microspheres from each batch was taken into size 2 capsule shell and transferred in each dissolution basket. The dissolution process was carried out for 8 hours and 10 mL dissolution sample was withdrawn at predetermined different time intervals and replaced with the same volume of test medium to maintain sink conditions. Each and every time 10 mL dissolution sample was compensated by fresh 10 mL of 0.1 N HCl. Dissolution samples were withdrawn with the help of 10 mL syringe and kept in test tube. The withdrawn samples were diluted, where necessary, filtered through 0.45*μ* membrane filter, and analyzed in UV-VIS spectrophotometer at wavelength of 210 nm.

### 2.6. Kinetic Analysis of Dissolution Data

To study the mechanism of drug release from the microspheres, the release data were fitted to zero order, first order, and Higuchi equation. Therefore, the dissolution data was fitted to the well-known exponential equation (Korsmeyer equation), which was often used to describe the drug release behavior from polymeric system [5]:

(2) $$\begin{array}{c} \log\left(\;\frac{Mt}{mf}\;\right)=\log K+\log t, \end{array}$$

where *Mt* is the amount of drug release after infinite time and *K* is release rate constant incorporating structural and geometric characteristics of the mechanism of drug release. To clarify the release exponent batches of microspheres, the log value of percentage drug dissolved was plotted against log time for each batch according to the above equation. A value of *n* = 0.45 indicates Fickian (case I) release; a value of >0.45 but <0.89 indicates non-Fickian (anomalous) release; and >0.89 indicates super case II type of release. Case II generally refers to the erosion of the polymeric chain and anomalous transport (non-Fickian) refers to combination of both diffusion and erosion controlled drug release.

### 2.7. Successive Fractional Dissolution Time

To characterize the drug release rate in different experimental conditionals like *T*_25%_, *T*_50%_, and *T*_80%_ they were calculated from dissolution data according to the following equations:

(3) T25%=0.25k1/n,T50%=0.50k1/n,T80%=0.80k1/n.

Another fractional tool MDT (mean dissolution time) can be calculated by the following equation:

(4) $$\begin{array}{c} MDT=\left(\;\frac{n}{n+1}\;\right)\cdot K^{-1/n}. \end{array}$$

MDT value is used to characterize the drug release rate from the microspheres and the retarding efficiency of the polymer. A higher value of MDT indicates higher drug retarding ability of the polymer and vice versa. The MDT value is also considered to be a function of polymer loading, polymer nature, and physico-chemical properties of the drug molecule [6].

### 2.8. Surface Morphology Study with the Help of Scanning Electron Microscope (SEM)

Surface nature of microspheres was examined with the help of Scanning Electron Microscope (JEOL, JSM-6490 LA, Japan). The microspheres were dried completely before examination and SEM was done at different magnifications of 20.0 kv × 75, 20.0 kv × 90, 20.0 kv × 95, 20.0 kv × 140, 20.0 kv × 300, 20.0 kv × 600, and 20.0 kv × 1000.

### 2.9. Fourier Transform Infrared (FTIR) Spectroscopy Studies

The FTIR technique is used to measure the absorption of various infrared radiations by the target material, to produce an IR spectrum that can be used to identify functional groups and molecular structure in the sample. FTIR spectra of pure Vildagliptin and formulated microspheres were recorded by using FTIR 8400S (SHIMADZU, Japan). Appropriate quantity of KBr and microspheres (in the ratio 100 : 2) was mixed by grinding in agate mortar. Disk was made with about 100 mg mixture under hydraulic pressure of 600 kg. Then the FTIR spectra were recorded between 4000 and 400 cm^−1^. The resolution was 2 cm^−1^.

### 2.10. Drug-Polymer Compatibility Study by Differential Scanning Calorimetry (DSC) Study of Microspheres

The DSC measurements were performed on DSC-60 (SHIMADZU) differential scanning calorimeter with thermal analyzer (TA-60WS). Pure Vildagliptin microsphere sample of 7.8 mg was placed in aluminum pan and sealed before heating under nitrogen flow (300 mL/min) at a scanning rate of 10°C min^−1^ from 30°C to 550°C. An empty aluminum pan was used as reference.

### 2.11. *In Vivo* Study

#### 2.11.1. Experimental Animals

At first Albino rats were taken (about 165 to 200 gm) and collected from the animal house of Jahangirnagor University and kept back in an area with a 12-hour day-night cycle, at even temperature of 22°C and humidity of 45–64%. During the investigational study rats were fed on pellets (Rat Feed, Bangladesh) with free access to distilled water.

#### 2.11.2. Induction of Experimental Diabetes

Rats were turned into diabetic ones via single intraperitoneal injection of freshly ready Streptozotocin (STZ-65 mg/kg body weight) in 0.1 M citrate buffer (pH 4.5) in volume of 1 mL/kg body weight. Standard rats received 1 mL citrate buffer as vehicle. After 48 hrs of Streptozotocin administration, blood glucose levels were estimated in rats following overnight fasting. Rats with blood glucose ranging between 200 and 300 mg/dL were considered diabetic and used for the experiments.

#### 2.11.3. Drugs and Chemicals

Streptozotocin was procured from Sigma Aldrich Co., St. Louis, MO, USA; 0.1 M citrate buffer (pH 4.5) was procured from Merck, Germany. All other biochemicals and chemicals used for the experiment were of analytical grade.

#### 2.11.4. Experimental Design

The rats were divided into 4 groups comprising 6 animals in each group as follows.

*Group I (Negative Control)*. Normal rats were treated with saline daily and served as the negative control.

*Group II (Positive Control)*. Animals were treated with single dose of Streptozotocin (65 mg/kg) by the intraperitoneal route to induce diabetes and served as a positive control.

*Group III (Pure Vildagliptin)*. Diabetic rats were treated with 2.5 mg/kg body weight of pure Vildagliptin.

*Group IV (Formulation VF13)*. Diabetic rats were treated with 2.5 mg/kg body weight of Vildagliptin containing microsphere.

#### 2.11.5. Collection of Blood from the Rats

After the experimental course of therapy, the rats were sacrificed by cervical dislocation under mild chloroform anesthesia. Blood was collected on decapitation and serum was separated by centrifugation (for 20 min at 2000 rpm).

#### 2.11.6. Estimation of Biochemical Parameters in Serum or Plasma

Serum glucose, cholesterol, triglycerides, urea, creatinine, bilirubin, and glycohemoglobin were assayed using diagnostic reagent kit manufactured by Crescent Diagnostics Ltd., Atlas Medical Diagnostics Ltd., and Stanbio Diagnostics Ltd.

## 3. Results and Discussions

### 3.1. Standard Curve Analysis

**Figure 2:**
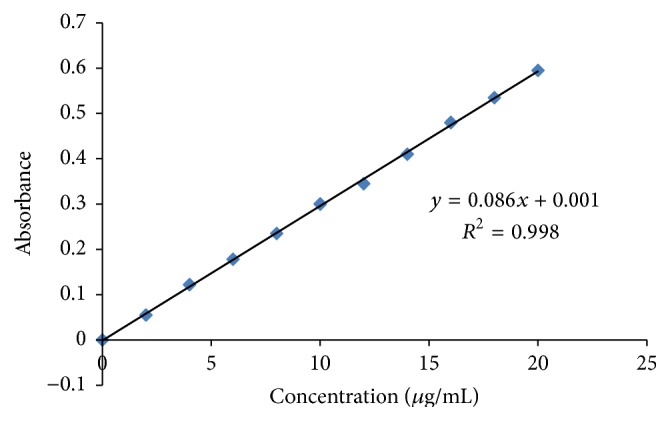
Standard curve of Vildagliptin.

Absorbance was taken at 210 nm with the help of UV-Visible spectrophotometer and the data were plotted in graph which has *y* = 0.086*x* + 0.001 and *R* ^2^ = 0.998 shown in Figure 2.

### 3.2. Actual Drug Loaded and Drug Entrapment Efficiency (DEE) of Prepared Microspheres by Emulsion Solvent Evaporation Technique

**Figure 3:**
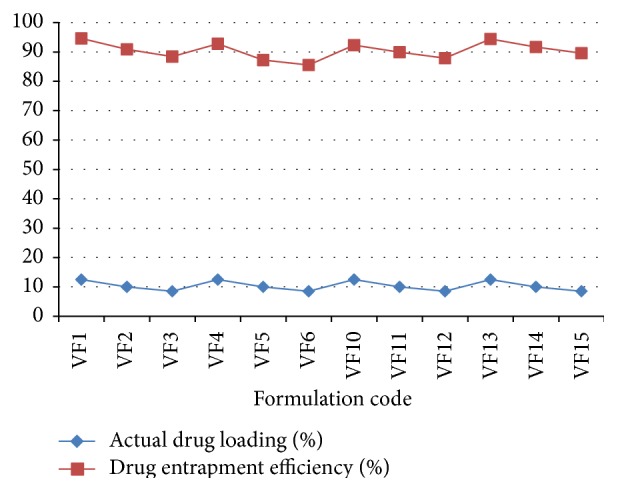
Comparative percent release study of actual drug loading and drug entrapment efficiency of different formulations.

Drug loading and the drug entrapment efficiency (DEE) of the prepared microspheres were carried out and the graphical presentations are given in Figure 3. The actual drug loaded and the drug entrapment efficiency were found to be in the range of 8.5% to 12.5% and 82.56% to 94.59%, respectively.

### 3.3. *In Vitro* Dissolution Study of Vildagliptin Microspheres Prepared by Emulsion Solvent Evaporation Technique

To find out the mechanism of drug release, the controlled release Vildagliptin microspheres were treated in different mathematical models like zero order (cumulative percentage of drug release versus time), first order (log percentage of drug remaining versus time), Higuchi model (cumulative percentage of drug release versus square root of time), and Korsmeyer model (log cumulative percentage of drug release versus log time). The release data was plotted. From the linear portions of the curve slope correlation coefficients (*R* ^2^) were calculated. With the Korsmeyer plot, linearity was noted highest in all formulations using all data points. The data yielded apparently straight line with Korsmeyer plot (*R* ^2^ > 0.99) but a bit with zero order, first order kinetics, and Higuchi plot. It is observed that drug released from sustained release microsphere followed Korsmeyer release log cumulative percentage of drug release versus log time. The mechanism of drug release was calculated according to Peppas equation. The calculated “*n*” values along with the correlation coefficients (*R* ^2^) have been shown in Table 2. The values of *n* depend upon the polymer concentration. The calculated “*n*” values suggest that the mechanism of drug release followed non-Fickian transport.

### 3.4. Effect of Different Polymers on the Release of Vildagliptin from Microspheres Prepared by Emulsion Solvent Evaporation Technique

**Figure 4:**
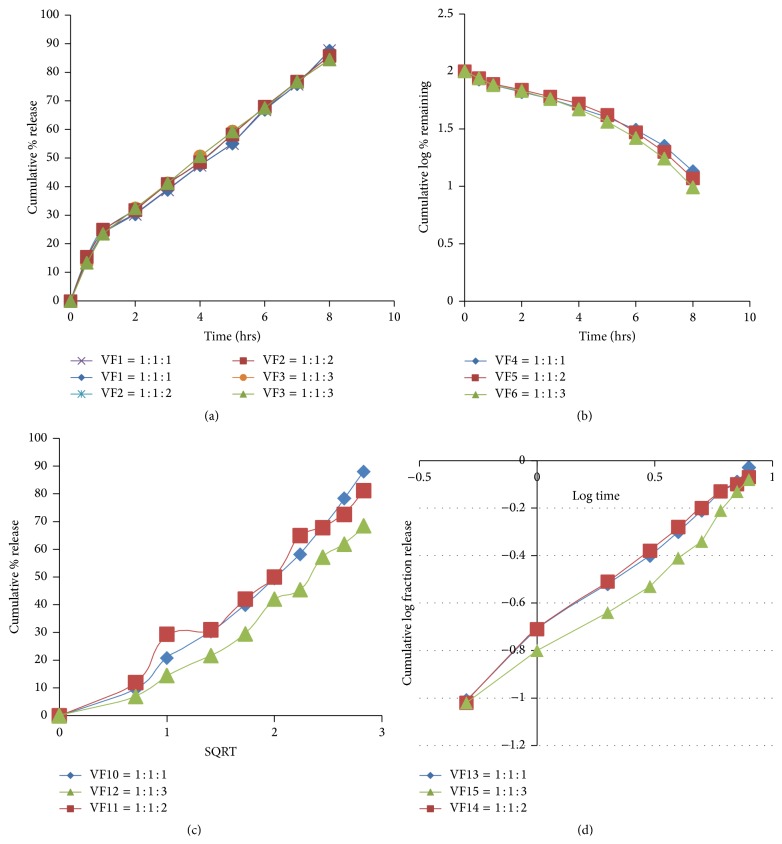
Release of Vildagliptin from microspheres prepared by emulsion solvent evaporation technique, respectively, where (a) zero order is from VF1 to VF3, (b) first order is from VF4 to VF6, (c) Higuchi model is from VF10 to VF12, and (d) Korsmeyer model is from VF13 to VF5.

Vildagliptin microspheres were prepared by polymeric concentration variation to study the effect of combination of polymers on the release of drug from microspheres. Formulations VF1 to VF3 were prepared by using Eudragit RS100 and Ethyl cellulose. After the end of 8 hours of dissolution, the release drug from microspheres was 87.5%, 85.7%, and 84.5%, respectively, which is shown in Figure 4(a). Formulations VF4 to VF6 were prepared by using Eudragit RL100 and Ethyl cellulose. After the end of 8 hours of dissolution, the release drug from microspheres was 86.6%, 88.2%, and 90.2%, respectively, which is shown in Figure 4(b). Formulations VF10 to VF12 were prepared by using Eudragit RS100 and Methocel K100M. After the end of 8 hours of dissolution, the release drug from microspheres was 88.0%, 81.1%, and 68.5%, respectively, which is shown in Figure 4(c). Formulations VF13 to VF15 were prepared by using Eudragit RL100 and Methocel K100M. After the end of 8 hours of dissolution, the release drug from microspheres was 92.4%, 85.5%, and 81.23%, respectively, which is shown in Figure 4(d).

It is noticeable that the entrapment efficiency of Eudragit RL100 microspheres was higher than that of the Eudragit RS100 microspheres. Eudragit RL100 contains higher amount of quaternary ammonium groups, which facilitates the diffusion of a part of entrapped drug to the surrounding medium during preparation of microspheres. Eudragit RS100 has thick polymeric surfaces due to the presence of lower amount of quaternary ammonium groups, which restrict the migration of drug particles to the surrounding medium. This suggested that the release of Vildagliptin from Eudragit RS100 microsphere exhibits diffusional characteristics, closely following Higuchi model, and is highly correlated with Korsmeyer-Peppas model release kinetics. This difference in drug release behavior suggested structural differences of the wall materials, and it is dependent on the content of the quaternary ammonium groups. Ethyl cellulose has given good retardant effect due to its hydrophobic nature, less permeability in dissolution medium by decreasing the drug diffusion. Methocel, on the other hand, due to its high water absorption ability and fast hydration and swelling might form an outer pseudo-gel layer to control drug release from the inner to the outer side of the microspheres. Thus the results showed that the release rate of drug from the microspheres can be modulated with adjusting the ratios of polymer/drug in the formulation.

All the formulations were best fitted with Korsmeyer model as shown in Table 2. The data obtained were also put in Korsmeyer-Peppas model in order to find out *n* value, which describes the drug release mechanism. The *n* value of microspheres of different drug to polymer ratio was ranged between 0.45 and 0.83, indicating that the mechanism of the drug release was diffusion controlled and erosion.

### 3.5. Comparative Study of Percent Release of Vildagliptin after 8 Hours of Different Formulations Prepared by Emulsion Solvent Evaporation Technique

**Figure 5:**
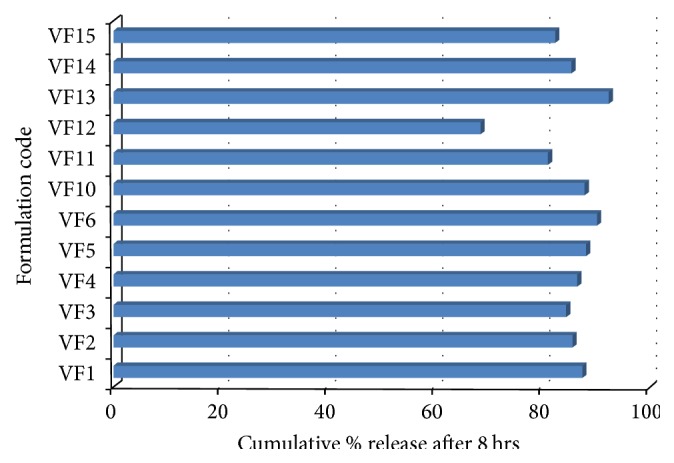
Comparative release studies of Vildagliptin microspheres after 8 hrs.

From Figure 5 it has been seen that drug entrapment efficiency of different formulations was in range of 82.56% to 94.59%. It has been seen that when percent of drug loading increased the percent of drug entrapment also increased for all formulations containing Eudragit RS100 and Ethyl cellulose (94.59%, 90.89%, and 88.43%). This tendency was found for other formulations containing Eudragit RL100 and Ethyl cellulose; Eudragit RS100 and Methocel K100M; Eudragit RL100 and Methocel K100M.

### 3.6. Successive Fractional Dissolution Time

**Figure 6:**
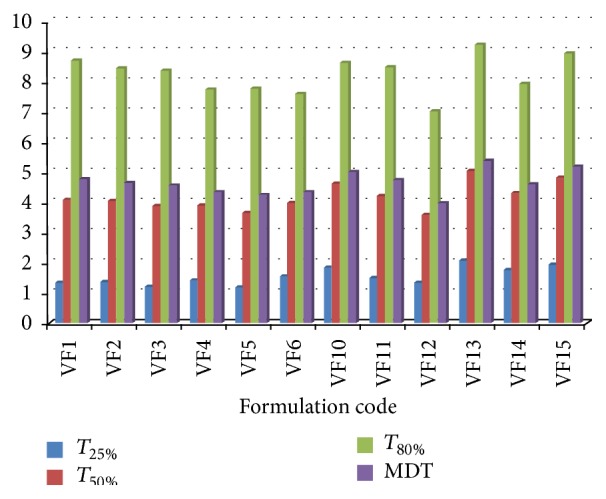
Successive fractional dissolution time (*T*_25%_, *T*_50%_, MDT, and *T*_80%_) of different formulations, respectively.

To characterize the drug release rate in different experimental conditionals they were calculated from dissolution data. MDT of formulations VF1, VF2, and VF3 were found 4.78 hours, 4.67 hours, and 4.58 hours, respectively. MDT of formulations VF4, VF5, and VF6 were found 4.36 hours, 4.28 hours, and 4.35 hours, MDT of formulations VF10, VF11, and VF12 were found 4.99 hours, 4.76 hours, and 3.98 hours, and MDT of formulations VF13, VF14, and VF15 were found 5.39 hours, 4.62 hours, and 5.18 hours, respectively, which is shown in Figure 6. The figure indicates that the higher the polymer level, the lower the value of *T*_25%_, *T*_50%_, MDT, and *T*_80%_, and they behave according to their properties.

### 3.7. Effect of Polymers Concentration on the Surface Morphology of Vildagliptin Microspheres Prepared by Emulsion Solvent Evaporation Technique

**Figure 7:**
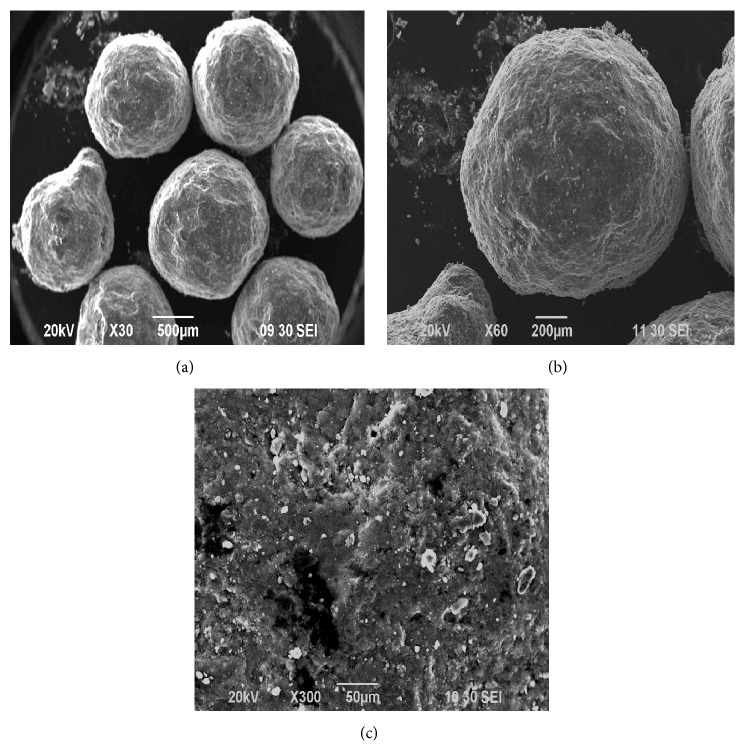
SEM studies of Vildagliptin microspheres of formulation VF13 prepared by emulsion solvent evaporation technique with different magnification (a) at ×30 SEI, (b) at ×60 SEI, and (c) at ×300 SEI, respectively.

SEM study showed that microspheres VF13 made of Eudragit RL100 and Methocel K100M were spherical and aggregated shown in Figure 7. The SEM of drug-polymer loaded microspheres had uneven shell owing to elevated application of drug in the microspheres. Surface analysis of the microspheres following liberate study showed larger pores signifying that the drug was unconfined from side to side pores and the means of drug release was diffusion controlled.

### 3.8. Drug-Polymer Compatibility Study by Fourier Transform Infrared (FTIR) Spectroscopy

**Figure 8:**
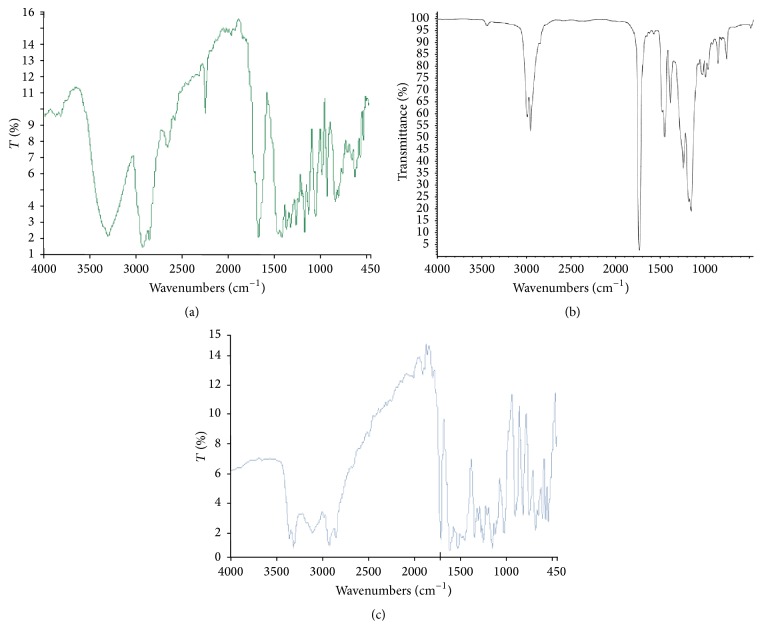
FTIR spectrum of (a) pure drug Vildagliptin, (b) Eudragit RL, and (c) formulation VF13, respectively.

The FTIR spectrum of pure drug showed characteristic amide peaks at 3367.5, 3314.3, and 1713.5 per cm, urea carbonyl stretching (urea N-H stretching) vibrations at 1618.4 and 1526.5 per cm, and SO_2_ stretching vibration at 1158 and 1341.5 per cm that are shown in Figure 8. There were no new bands observed in the spectrum, which confirms that no new chemical bonds were formed between the drug and the polymer.

### 3.9. Drug-Polymer Compatibility Study by Differentiate Scanning Calorimetry (DSC) Study of Microspheres

**Figure 9:**
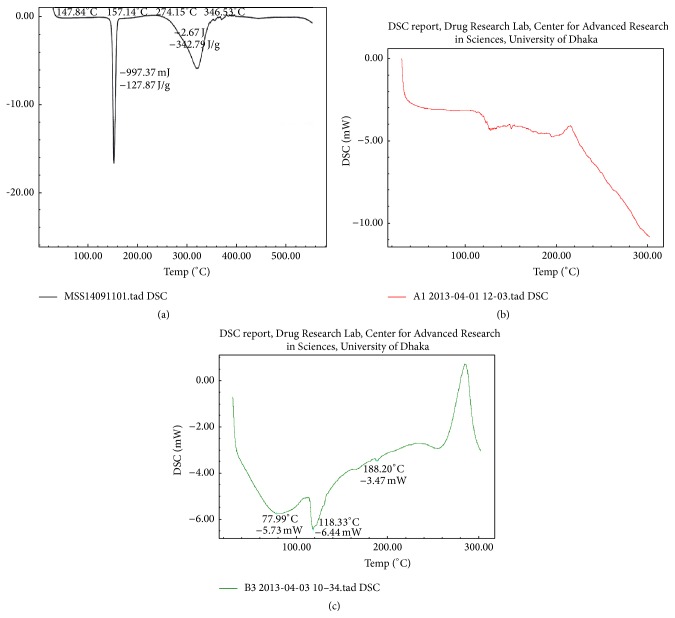
DSC of (a) pure Vildagliptin, (b) Eudragit RL100, and (c) formulation VF13.

DSC is a fast and reliable method for understanding polymorphic transitions when screening drugs and polymers for compatibility, obtaining information about possible interactions. It was evident from the DSC profile (Figure 9) that Vildagliptin exhibited a sharp endothermic peak at 172.99°C, which corresponds to the drug crystallinity whereas formulation VF13 is thrashing its sharpness at its endothermic peak at 118.33°C. It appears that there is a significant reduction of drug crystallinity in the microspheres. The absence of detectable crystalline domains in drug loaded microspheres clearly indicates that drug was dispersed completely in the formulation, thus modifying the microspheres to an amorphous, disordered-crystalline phase.

### 3.10. Study of* In Vivo* Antihyperglycemic and Different Biochemical Effects on Albino Rats

From Table 3 it was observed that Streptozotocin could induce diabetics greatly in rats. On the other hand pure drug reduced the blood glucose level in an irregular manner up to 7 hours and then blood glucose level increased to the top, because it could not give sustained release of drug. It might be due to the fact that, here, Vildagliptin rapidly inhibited DPP-4 activity which increased fasting and postprandial endogenous levels of the incretin hormones GLP-1 (glucagon-like peptide 1) and GIP for short time and reduces blood glucose level rapidly. But the drug release was not sustained for a long time uniformly due to the absence of sustained release polymers.

For the formulation VF13 it was observed that it provided sustained release of drug up to 08 hours and that was proved by the data of reduction of blood glucose level in Table 3. Here administration of sustained release formulation of Vildagliptin resulted in a rapid and complete inhibition of DPP-4 activity, resulting in increased fasting and postprandial endogenous levels of the incretin hormones GLP-1 (glucagon-like peptide 1) and GIP. As a result blood glucose level reduced rapidly and reduced glucose level maintained for 08 hours smoothly. It was due to the uniform sustained release of drug from formulations containing sustained release polymers Methocel K100M and Eudragit RL100.

**Figure 10:**
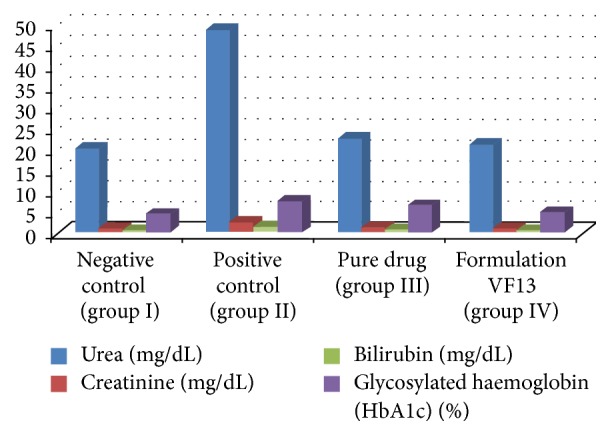
Effect of different formulation on urea, creatinine, bilirubin, and glycosylated haemoglobin (HbA1c) in serum or plasma of control and experimental groups.

**Figure 11:**
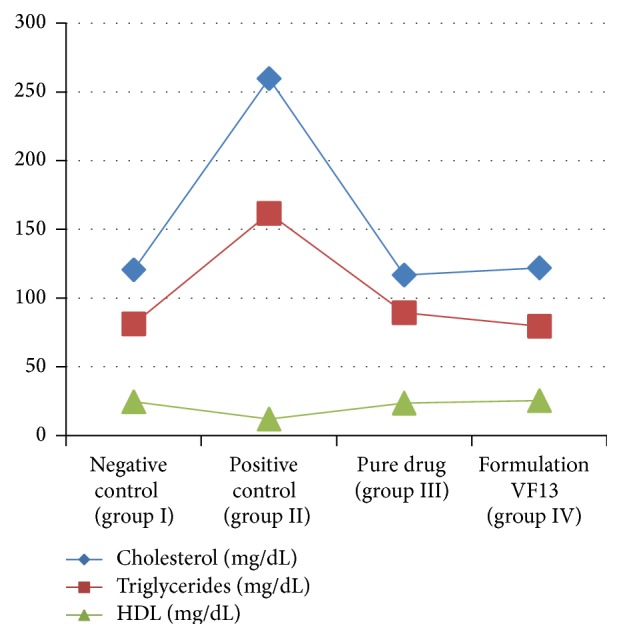
Effect of drug on lipid profile of control and experimental groups.

STZ diabetic rats were found to have significantly elevated serum creatinine and urea levels as compared to nondiabetic control rats. This is because STZ diabetic rats have diminished ability to filter urea and creatinine from blood and excrete them in urine. This is another characteristic change in diabetes. Whereas after treatment both the values were comparable to those which received Vildagliptin treatment, there was no significant difference in values obtained for group I and group IV shown in Figure 10. Various parameters of blood lipid profiles were tested in the normal and diabetic rats. The levels of TC and TG were significantly elevated and level of serum HDL was decreased in diabetic control group as compared to normal control rats. In case of insulin deficiency, there is increased lipolysis leading to hyperlipidemia. In insulin deficient diabetes, the concentration of free fatty acids is elevated as a result of free fatty acid outflow from fat depots, where the balance of free fatty acid esterification-triglyceride lipolysis cycle is displaced in favor of lipolysis. It has been shown from Figure 11 that, after being treated with formulation VF13, the alteration in lipid metabolism was partially attenuated as evidenced by decreased serum TG and TC levels in diabetic rats.

## 4. Conclusion

The present study was conducted to design Vildagliptin sustained release microspheres by emulsion solvent evaporation technique.* In vitro* dissolution study showed the sustained release of Vildagliptin from the microsphere for about 8 hours. From the* in vitro* dissolution data it has been established that the drug dissolution profile could be slowed down by increasing the amount of retardant polymers in the formulations and 5 : 5 solvent ratios ensured the better sustained release. Scanning electron microscopy showed the smooth and slightly porous surface of microspheres. From differential scanning calorimetric test it can be concluded that there was no significant change in melting point and glass transition temperature and no significant crystallinity. FTIR data showed absence of any new functional group and any other interaction in between drugs and polymers. In addition formulated microspheres can be chosen for* in vivo* antidiabetic study and another biomedical study exhibited satisfactory results.

So from the results it can be concluded that drug retardant polymers and their concentration affect all the evaluation parameters significantly. Hence the prepared polymeric microspheres of Vildagliptin might be proved to be potential candidate for safe and effective sustained drug delivery from microspheres for the treatment of type II diabetes.

## Figures and Tables

**Table 1:** Formulation of Vildagliptin microspheres prepared by emulsion solvent evaporation technique.

Formulation code	Polymers (mg)			
	D : EURS100 : EC	D : EURL100 : EC	D : EURS100 : MK100M	D : EURL100 : MK100M
VF1	1 : 1 : 1	—	—	—
VF2	1 : 1 : 2	—	—	—
VF3	1 : 1 : 3	—	—	—
VF4	—	1 : 1 : 1	—	—
VF5	—	1 : 1 : 2	—	—
VF6	—	1 : 1 : 3	—	—
VF10	—	—	1 : 1 : 1	—
VF11	—	—	1 : 1 : 2	—
VF12	—	—	1 : 1 : 3	—
VF13	—	—	—	1 : 1 : 1
VF14	—	—	—	1 : 1 : 2
VF15	—	—	—	1 : 1 : 3

D = drug, EC = Ethyl cellulose, EU = Eudragit, and M = Methocel.

**Table 2:** Release rate constants and correlation coefficient (*r* ^2^) values of different formulations of Vildagliptin microspheres using different polymers by emulsion solvent evaporation technique, respectively.

Formulation code	Rate constants and *R*-squared values							
	Zero order		First order		Higuchi		Korsmeyer-Peppas	
	*K*_0_	*R* ^2^	*K*_1_	*R* ^2^	*K*_*H*_	*R* ^2^	*n*	*R* ^2^
VF1	9.83	0.983	−0.218	0.919	29.81	0.96	0.62	0.991
VF2	9.73	0.979	−0.211	0.951	29.7	0.974	0.61	0.995
VF3	9.8	0.981	−0.209	0.969	30.06	0.98	0.64	0.994
VF4	9.97	0.979	−0.221	0.929	30.57	0.98	0.63	0.994
VF5	10.35	0.988	−0.211	0.957	31.33	0.963	0.67	0.991
VF6	10.67	0.984	−0.204	0.964	32.54	0.973	0.66	0.993
VF10	10.4	0.989	−0.225	0.925	31.51	0.966	0.75	0.991
VF11	9.39	0.951	−0.221	0.944	29.16	0.976	0.67	0.995
VF12	8.48	0.988	−0.218	0.965	25.54	0.959	0.7	0.994
VF13	11.13	0.993	−0.222	0.945	33.51	0.958	0.78	0.995
VF14	10.6	0.981	−0.225	0.954	32.37	0.973	0.77	0.995
VF15	9.86	0.987	−0.231	0.924	29.05	0.912	0.76	0.992

**Table 3:** Average reduction in glucose level (mg/dL) in negative control, positive control, pure drug Vildagliptin, and formulation VF13.

Time (hour)	Average glucose level reduction (mg/dL)			
	Negative control (group I)	Positive control (group II)	Pure drug (group III)	Formulation VF13 (group IV)
0	70 ± 5.329	311.9 ± 5.92	361.44 ± 5.44	344.17 ± 7.49
1	70 ± 5.556	312.3 ± 4.36	205.82 ± 13.25	233.17 ± 8.68
2	70.1 ± 5.663	316.16 ± 7.77	157.7 ± 11.26	214.87 ± 7.71
3	70.2 ± 3.445	318.77 ± 8.67	137.4 ± 9.89	202.75 ± 8.21
4	70.25 ± 3.687	325.53 ± 11.26	110.08 ± 10.94	174.75 ± 5.94
5	70.29 ± 3.567	329.33 ± 3.77	119.98 ± 8.90	140.07 ± 4.67
6	70.3 ± 2.478	329.83 ± 4.78	139.96 ± 7.37	118.92 ± 3.32
7	70.3 ± 3.568	335.57 ± 10.57	162.32 ± 10.81	108.32 ± 3.70
8	70.31 ± 3.598	338.9 ± 11.93	186.14 ± 11.03	102.37 ± 4.68
12	71 ± 2.456	351.77 ± 12.06	237.56 ± 9.66	132.72 ± 5.19
24	71 ± 1.456	362.77 ± 7.39	307.68 ± 7.97	279.82 ± 6.05
48	71.2 ± 2.786	392.77 ± 7.40	317.68 ± 9.98	309.82 ± 8.06

Values are given as mean ± standard deviation for group of six rats.
